# Assessing water stress in a high-density apple orchard using trunk circumference variation, sap flow index and stem water potential

**DOI:** 10.3389/fpls.2023.1214429

**Published:** 2023-08-03

**Authors:** William D. Wheeler, Brent Black, Bruce Bugbee

**Affiliations:** ^1^ Crop Physiology Laboratory, Department of Plants, Soils, and Climate, Utah State University, Logan, UT, United States; ^2^ Pomology Extension, Department of Plants, Soils, and Climate, Utah State University, Logan, UT, United States

**Keywords:** band dendrometer, sap flow, stem water potential, water stress, Fuji, Scilate, high density, dwarfing rootstocks

## Abstract

**Introduction:**

Automated plant-based measurements of water stress have the potential to advance precision irrigation in orchard crops. Previous studies have shown correlations between sap flow, line variable differential transform (LVDT) dendrometers and fruit tree drought response. Here we report season-long automated measurement of maximum daily change in trunk diameter using band dendrometers and heated needles to measure a simplified sap flow index (SFI).

**Methods:**

Measurements were made on two apple cultivars that were stressed at 7 to 12 day intervals by withholding irrigation until the average stem water potential (Ψ_Stem_) dropped below -1.5 MPa, after which irrigation was restored and the drought cycle repeated.

**Results:**

Dendrometer measurements of maximum daily trunk shrinkage (MDS) were highly correlated (r² = 0.85) with pressure chamber measurements of stem water potential. The SFI measurements were less correlated with stem water potential but were highly correlated with evaporative demand (r² = 0.82) as determined by the Penman-Monteith equation (ET_r_).

**Discussion:**

The high correlation of SFI to ET_r_ suggests that high-density orchards resemble a continuous surface, unlike orchards with widely spaced trees. The correlations of MDS and SFI to Ψ_Stem_ were higher during the early season than the late season growth. Band dendrometers are less labor intensive to install than LVDT dendrometers and are non-invasive so are well suited to commercialization.

## 1 Introduction

Commercial apple production in many areas of the world is almost exclusively accomplished through grafting of genetically different fruit bearing scions to well adapted rootstocks. Newly established orchards have shifted toward dwarfing rootstocks and high planting densities (Robinson, 2006). Furthermore, one of the most common and widely planted apple rootstocks is M.9 due to its highly dwarfing nature, precociousness and high fruit set (Fallahi et al., 2002). Dwarfing rootstocks have reduced root volumes, which makes them more prone to drought stress and necessitates careful irrigation management (Gonçalves et al., 2006). In pome fruit, regulated deficit irrigation during fruit development has been found to increase fruit number and soluble solids content while decreasing fruit size (Marsal et al., 2002). When deficit irrigation was applied during vegetative growth, vigorous shoot growth and trunk expansion was suppressed (Ebel et al., 1995). Without precise regulation of this irrigation deficit however drought can quickly reduce yields and can lead to tree mortality. Conversely, over application of irrigation can promote excessive vegetative growth, increased pathogen pressure and leach nutrients from the rootzone (Bonany and Camps, 1996). Precise understanding of tree water status can inform irrigation timing, ensure tree health and maximize productivity. An accurate and cost-effective method that can be easily implemented in the field is urgently needed to help orchard producers advance precision irrigation methodology and maximize yields

Evapotranspiration (ET) modeling is a widely used tool for estimating crop water loss in commercial orchard management to estimate tree water status. Numerous models exist to compute potential evapotranspiration (ET) (e.g. Priestley and Taylor, 1972 (Priestley and Taylor, 1972), Hargreaves and Samani, 1985 (Hargreaves and Samani, 1985), Shuttleworth and Wallace, 1985 (Shuttleworth and Wallace, 1985)); the most widely used is the FAO - 56 Penman-Monteith equation. Reference evapotranspiration values for a grass (ET_0_) or alfalfa-like (ET_r_) crop are commonly reported from weather stations and are then used in conjunction with empirically derived crop coefficients to estimate specific crop water losses. While this methodology is effective in many annual crops, results in orchard crops have been mixed (Naor et al., 2008; Dzikiti et al., 2018). The height of orchard trees and low planting densities compared to those of reference crops have been cited as reasons for divergence of orchard ET from modeled ET with modifying crop coefficients (Jarvis, 1984). For these reasons, the use of reference models as an accurate predictor of tree water status has been questioned (Annandale and Stockle, 1994).

Soil moisture measurements are a relatively inexpensive and intuitive method for controlling irrigation in commercial orchards but have several limitations. Soil moisture is an indirect measure of tree hydration, and tree hydration is determined by both soil water availability and environmental demand (Jones, 2004). Moreover, soil heterogeneity and the extensive spread of tree roots mean that soil moisture availability can vary greatly within an orchard, making it necessary to use a large number of soil moisture sensors to capture this variability (Pardossi et al., 2009).

Direct plant-based measurements of water stress have long been considered the best approach for automating irrigation in orchard crops (Jones, 2004). Midday stem water potential (Ψ_Stem_) is considered a reliable indicator of peak water stress for fruit trees (Naor et al., 1995; Doltra et al., 2007), but the use of Scholander type pressure chambers to determine Ψ_stem_ is labor intensive, time consuming and cannot easily be automated. To achieve accurate deficit irrigation, orchard managers need plant-based measurements that are easily automated and interpreted.

Sap flow sensors can provide a direct, near instantaneous method for measuring sap flow, which is highly correlated with tree transpiration (Burgess et al., 2001). However, calibrating these types of sensors for absolute values is complex and prone to error, even for experienced researchers (Forster, 2017). Relative values of heat velocity and sap flow are well correlated to environmental demand and transpiration (Burgess and Dawson, 2007; Ballester et al., 2012; Forster, 2017). If the primary objective is to analyze and leverage sap flow responses to biotic or abiotic stressors, sap flow sensors can be used to estimate relative transpiration without extensive calibration.

Diurnal trunk diameter variation has also been proposed as an automated measure of plant water status for irrigation scheduling (Goldhamer and Fereres, 2001). During the night, the stem rehydrates and its diameter reaches its maximum near sunrise. Stem diameter then contracts during the day and reaches its minimum diameter a few hours after solar noon when evaporative demand is highest (Ginestar and Castel, 1995). The difference between the maximum and minimum trunk diameters in a 24-hour period is referred to as the maximum daily shrinkage (MDS) and is well correlated with Ψ_stem_ (Fernández and Cuevas, 2010). However, the use of point dendrometers, which are sensitive to position on the tree, has limited the effectiveness of dendrometers for irrigation scheduling due to the high degree of variability between measurements (Ortuño et al., 2010). Band dendrometers, on the other hand, measure changes in trunk circumference and can minimize position errors (Corell et al., 2014). Although widely used in forestry, band dendrometers have seen limited use in horticulture applications.

We sought to evaluate the effectiveness of a simplified relative sap flow index (SFI) and trunk circumferential fluctuations from band dendrometers as indicators for tree water status and irrigation scheduling in high density apple plantings. We investigated the correlation between atmospheric evaporative demand and sensor readings compared to midday Ψ_Stem_. We hypothesized that declining SFI values would be strongly correlated with decreasing Ψ_Stem_, indicating water stress. Additionally, we expected to observe a strong correlation between MDS from band dendrometers and Ψ_Stem_.

## 2 Materials and methods

### 2.1 Site description

Research was conducted at the Utah State University Research Farm located in Kaysville, UT (41° 01’ 21” N by 111° 55’ 51”W, elevation 1325 m) during the 2020 growing season (5/9/2020 – 10/7/2020). The region has a semi-arid continental climate under the Köppen classification system with average annual rainfall of 380 mm and an annual average pan evapotranspiration is 995 mm (57-year average). Sampling was done in a 0.5 ha, 6^th^ leaf, high density apple (*Malus* x *domestica* Borkh.) planting, with 1.5 m in row spacing and approximately 3 m between rows oriented north to south. Trees were trained to a tall spindle system with trunk diameters averaging 6-7 cm, 30 cm above the soil surface (Robinson et al., 2008). The orchard was originally established to examine the potential relationship between initial graft union strength and subsequent drought tolerance (Adams, 2016) and these goals ran concurrently with our trial. For this study two scion and rootstock combinations consisting of fruiting scions ‘Scilate’ (Envy™) (Scilate) (White, 2009) and ‘Aztec Fuji’ (Fuji) grafted with M.9 rootstocks were planted in blocks of six trees and replicated four times in a randomized complete block design.

**Figure 1:**
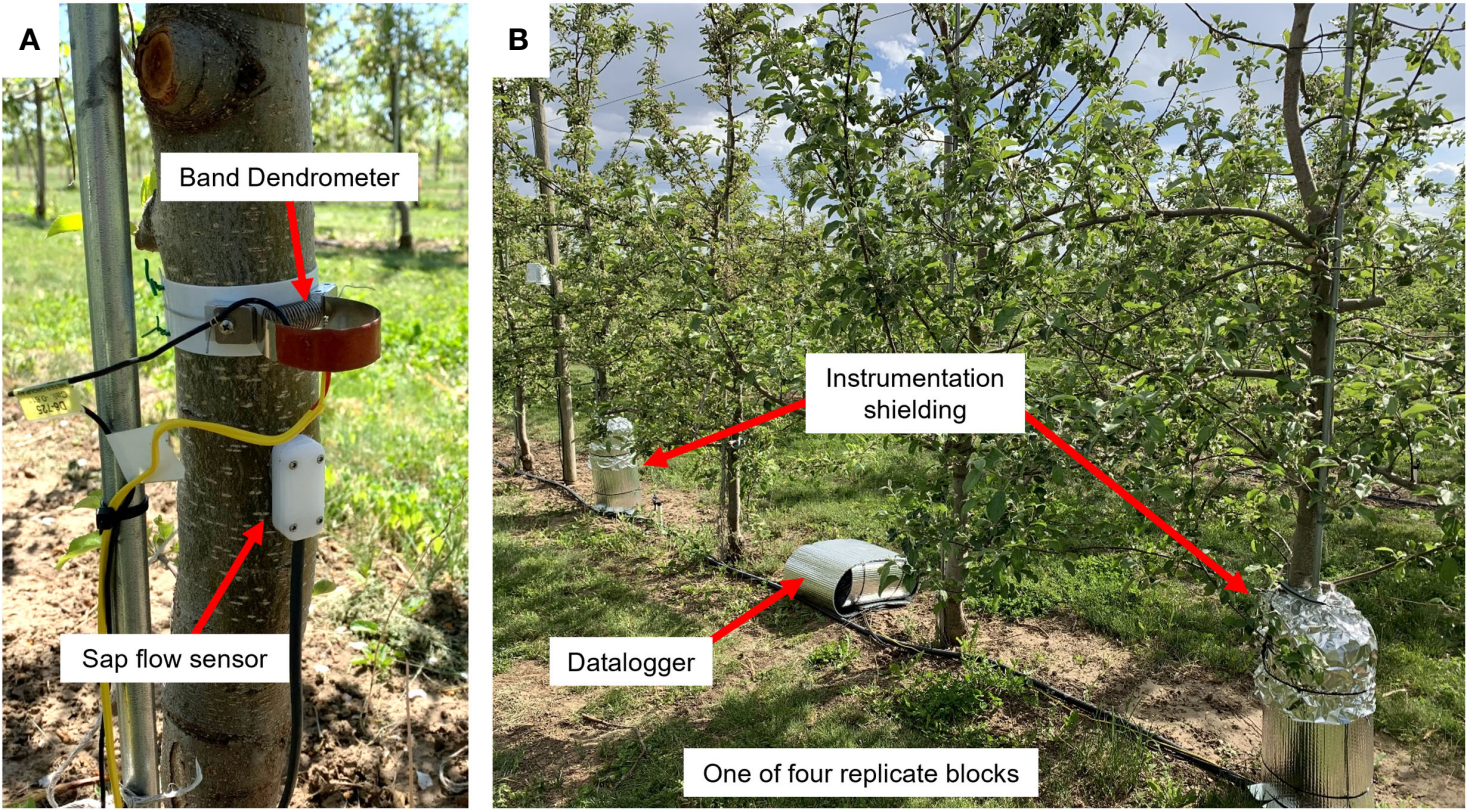
Representative experimental setup in a high-density apple block in which ‘Aztec Fuji’ and ‘Scilate’ (Envy™) fruiting scions were grafted to Malling 9 Selection NICTM 29 rootstocks. **(A)** A band dendrometer (above) and sap flow sensor (below) were installed 0.5 m above the soil surface, above the graft junction and were continuously monitored throughout the trial. **(B)** One of four dataloggers with insulation (center box) connected to two sensor instillations with thermal and protective shielding (left and right of center box).

To investigate drought responses irrigation was withheld from the entire plot until midday Ψ_stem_ from an average of twenty randomly selected trees dropped below -1.5 MPa. Then, between 20 and 80 mm of irrigation water was applied to recharge soil water. Initial applications of irrigation water were around 20 mm (~6 hrs run time) based on historical practice. However, this was deemed insufficient for deep soil recharge and irrigation was increased to 50 to 80 mm (~24 hrs cumulative run time) for subsequent applications. Trees were irrigated with micro-spray emitters with a 2 m overlapping spray radius with an approximate application rate of 3.4 mm hr^-1^. Over the course of the study, three irrigation events were initiated to return trees to non-drought stressed conditions, in addition to precipitation events that occurred mostly in the beginning of the season and naturally brought trees out of drought conditions. Soils were a well-drained Kidman series fine sandy loam. Four CR1000 dataloggers (Campbell Scientific, Logan UT, USA) were used to collect data from sap flow, dendrometer, and soil moisture sensors (**Figure 1**).

### 2.2 Stem water potential

Stem water potential (Ψ_Stem_) was measured twice weekly using a Scholander pressure chamber (Model 610; PMS Instrument Company, Albany, OR, USA). Three fully expanded leaves located near the main trunk of one tree per block (n=4) were covered with mylar bags for at least 2 hours before excision and measurement. Readings from the 3 leaves were averaged per tree and used in statistical analysis. Measurements were taken during midday (12:00 – 14:00) to ensure consistency and minimize the effects of diurnal variation.

### 2.3 Soil moisture

Soil water content was measured using dielectric sensors (GS3; Decagon Devices, Inc., Pullman, WA) installed between replicated plots (n=4). Readings were used to represent soil moisture for both Scilate and Fuji scions in each replicate plot. Sensors were buried one meter into the row at depths of 20 cm and 80 cm. The Topp equation (Topp et al., 1980) was used to convert dielectric permittivity volumetric water content (θ). Soil water measurements were used to evaluate the effectiveness of irrigation treatments and their impact on plant water status.

### 2.4 Sap flow index

Sap flow index (SFI) was measured using three-needle heat-pulse sensors (East 30 Sensors; Pullman, WA). Needles were made of stainless steel and were 1.2 mm in diameter, 35 mm long and spaced 6 mm apart. The outermost needles contained three precision thermistors, located at 5 mm, 17.5 mm and 30 mm from the needle base. For this study the thermistors located at 30 mm were not used as they were in the non-conducting heartwood. The innermost needle housed a 45 Ω nichrome wire heater, excited with 12 V for 8 sec every half hour. Individual trees within the blocks were selected to accommodate sensor cable lengths with one tree instrumented per block (n=4). A drill guide was used during installation to ensure accurate spacing and prevent probe misalignment. Sensors were placed approximately 0.5 m from the soil surface, above the graft junction and below the lowest branches; care was taken to avoid knots and deformities (**Figure 1**).

Heat velocity was determined using the dual method approach to resolve low and high rates of flow as suggested by Forster (2020) (Forster, 2020). Briefly, the dual method approach utilizes the Péclet equation to transition between the heat ratio method (Burgess et al., 2001) and temperature maximum method (Cohen, 1991) based on whether conduction or convection is the dominant process of heat transfer. Thermal diffusivity was assumed to be 0.0023 cm^2^ s^-1^ based on previously reported values for apple (Forster, 2020). Measurements were made every 30 min and averaged hourly and daily. A polynomial wounding correction was applied to measurements based on a 1.7 mm drill diameter (Burgess et al., 2001). Average daily wound corrected heat pulse velocity measurements provided an index of sap flow and used in all statistical analyses.

### 2.5 Trunk circumferential variation

Tree trunk circumferential variation was measured using band dendrometers (D6; UMS, Munich, Germany). Maximum daily shrinkage was calculated by the difference in a 24-hour period between the maximum and minimum trunk circumference. Maximum daily trunk circumference was determined once a day from the maximum circumference measurement that occurred between midnight and noon. Daily trunk growth rate (TGR) was calculated from the change in the maximum daily circumference from one day to the next (TGR = max. circumference day (n+1) – max. circumference day (n)). Circumferential growth patterns were analyzed by normalizing ending dendrometer voltages to final average scion circumferences. Dendrometers were installed approximately 0.5 m above the soil surface just above sap flow sensors on the North side of the tree (**Figure 1**). A cable made of Invar steel, which has an expansion coefficient close to zero, was used to secure the dendrometer around the stem (Katerji et al., 1994). Teflon mesh was placed between the sensor apparatus and tree trunk to allow the dendrometer to smoothly expand and contract diurnally and slowly expand to accommodate trunk growth over the growing season. Both the band dendrometer and sap flow sensors were shielded using a ridged metal frame that was insulated to minimize thermal loading.

### 2.6 Harvest and growth measurements

Fruit was harvested on day of the year (DOY) 281 and total harvest weight and crop load were determined per tree (n= 24). Average fruit size was calculated from the harvest weight and number of fruit per tree. Final trunk circumferential measurements were taken 30 cm above the soil surface for all trees using a flexible tape measure. Trunk cross sectional area (TCSA) was computed from these measurements. Stem elongation measurements were taken from three shoots per tree (n = 24), measuring from the last year’s growth to the tip of the new shoot.

### 2.7 Environmental measurements

Environmental data were collected by a weather station maintained by the Utah Climate Center located approximately 0.25 km to the southwest of the block. Sensors included a propeller blade and vane wind sensor (Model 05103, R.M. Young, Traverse City MI, USA), temperature/humidity probe (EE08, E+E Electronik, Engerwitzdorf, Austria), solar pyranometer (SP-230, Apogee Instruments, Logan UT, USA), and tipping bucket rain gage (TE525, Texas Electronics, Dallas TX, USA). Alfalfa reference evapotranspiration was estimated from these data using the American Society of Civil Engineers standardized reference ET_r_ equation (Allen et al., 2005).

### 2.8 Statistics

Four replicate blocks per scion were instrumented with sap flow sensors (n =4) and three blocks per treatment were instrumented with band dendrometers (n = 3). With the exception of one replicate block where only sap flow sensors were used, the same trees were instrumented with both sap flow and band dendrometers and Ψ_Stem_ was collected from the instrumented trees. When analyzing correlations to Ψ_Stem_, data were adjusted to reflect sample sizes of sap flow sensors and band dendrometers. Harvest, final trunk circumference, and stem elongation data were analyzed from all blocks in the plot. Data were separated into “early” and “late” seasonal responses based on understanding of phenological stages and analysis of circumferential growth (Liu et al., 2012). Late season responses were judged to begin at day of the year 185 based on plateauing of Fuji circumferential growth which corresponded roughly with date of the end of spur leaf expansion and beginning of fruit development. Sap flow index, MDS and Ψ_Stem_ were examined for correlations to environmental variables using linear regression. Sap flow index and MDS were also examined for correlations to Ψ_Stem_ using linear regression. Differences between grafted scions over the course of the season in SFI, MDS and Ψ_Stem_ were determined utilizing a linear mixed effects regression. A multiple linear regression model was used to identify the relationship between independent environmental variables and SFI. Statistical analysis was conducted using R statistical software (R Foundation for Statistical Computing, Vienna, Austria).

## 3 Results

### 3.1 Environmental conditions

**Figure 2:**
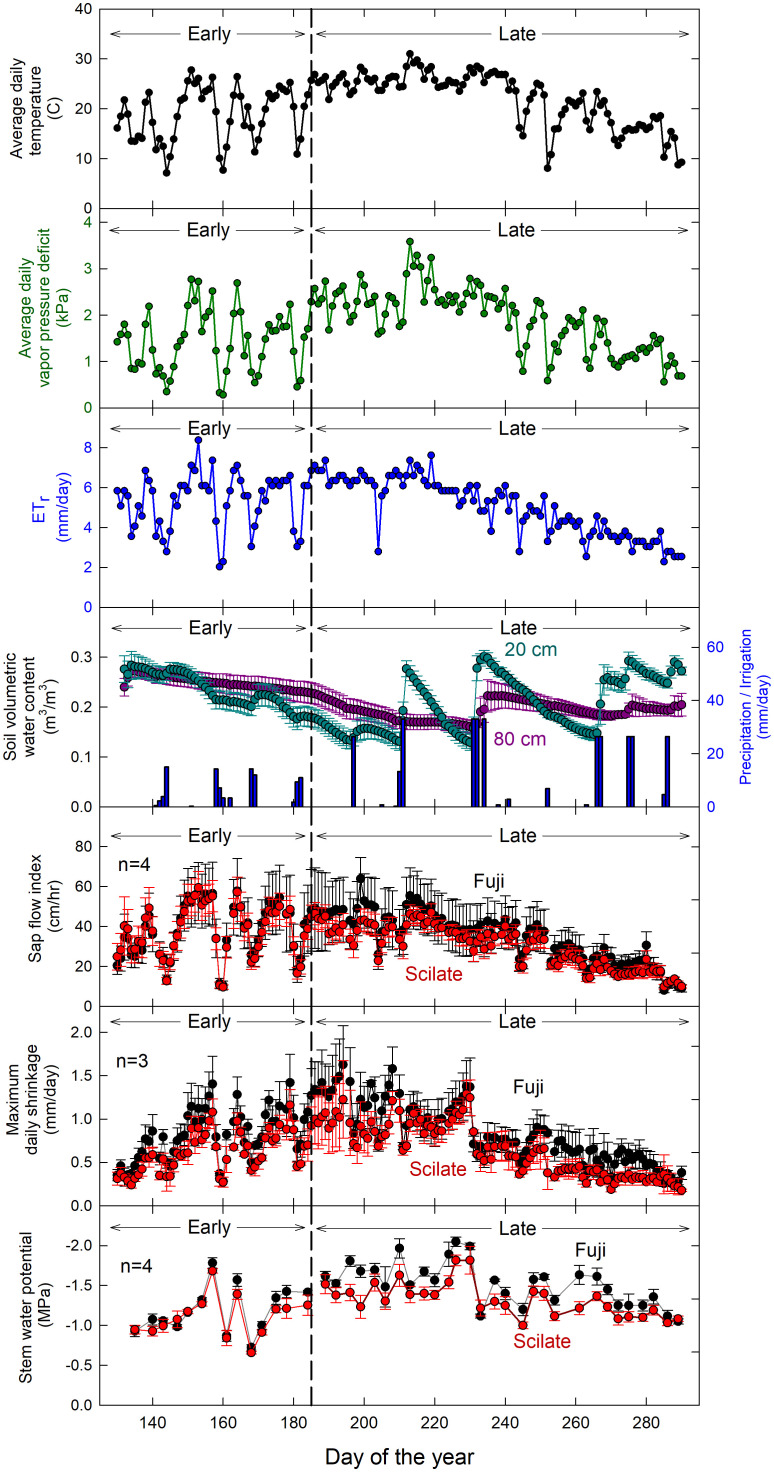
Time series of environmental data over the trial. Data for average air temperature (T_a_), vapor pressure deficit (VPD), evapotranspiration (ETr) and precipitation/irrigation collected by a weather station approximately 0.25 km away from the experimental apple block. This was compared to maximum daily shrinkage (MDS) (n=3), sap flow index (SFI) (n=4) and stem water potential (Ψ_stem_) (n=4). Lighter colors used for MDS, SFI and Ψ_Stem_ denote early season data while darker colors denote late season data. Error bars represent standard error.

Daily averages for the most significant environmental variables: air temperature (T_a_), vapor pressure deficit (VPD), alfalfa reference evapotranspiration (ET_r_), and soil moisture (θ) readings at 20 cm and 80 cm below the soil surface are shown in **Figure 2**. Average daily air temperature was 21.9°C and ranged from 7.1 to 31°C. Vapor pressure deficit averaged daily over the season was 1.85 kPa and ranged from 0.28 to 3.58 kPa. Correlations of physiological measurements to average daily VPD were compared to maximum observed VPD, and VPD averaged two hours before and after solar noon. This restricted time interval VPD averaging (e.g. daylight hours only, mid-day hours only) did not improve correlations compared to average daily VPD. Average daily ET_r_ was 5.47 mm d^-1^ over the season and ranged between 2.03 and 8.38 mm d^-1^. Rain fall occurred primarily in the beginning of the season and rainfall values totaled 97 mm over the course of the trial. There were 13 irrigation events over the course of the trial, accounting for approximately 555 mm of applied water. Soil moisture 20 cm below the surface of the soil averaged 0.21 m^3^ m^-3^ and ranged from 0.09 to 0.34 m^3^ m^-3^ while θ-80 cm below the soil surface averaged 0.22 m^3^ m^-3^ and ranged from 0.13 to 0.29 m^3^ m^-3^. Daily wind speed averaged 1.8 m s^-1^ over the course of the trial and solar radiation (R_n_) averaged 24.9 MJ m^-2^ d^-1^ ranging from 9.4 to 31.7 MJ m^-2^ d^-1^. There was a severe weather event that occurred on DOY 252 during which gusts of wind around 40 m s^-1^ were recorded, equivalent to wind speeds of that of a category 2 hurricane. As a result of this severe weather event, several trees (8) were uprooted and a significant portion (>50%) of fruit was blown from the trees. Fortunately, none of the instrumented trees were uprooted. Fruit loss was judged to have affected both scions similarly and harvest data was deemed useful for analysis.

### 3.2 Trunk circumferential variation

**Figure 3:**
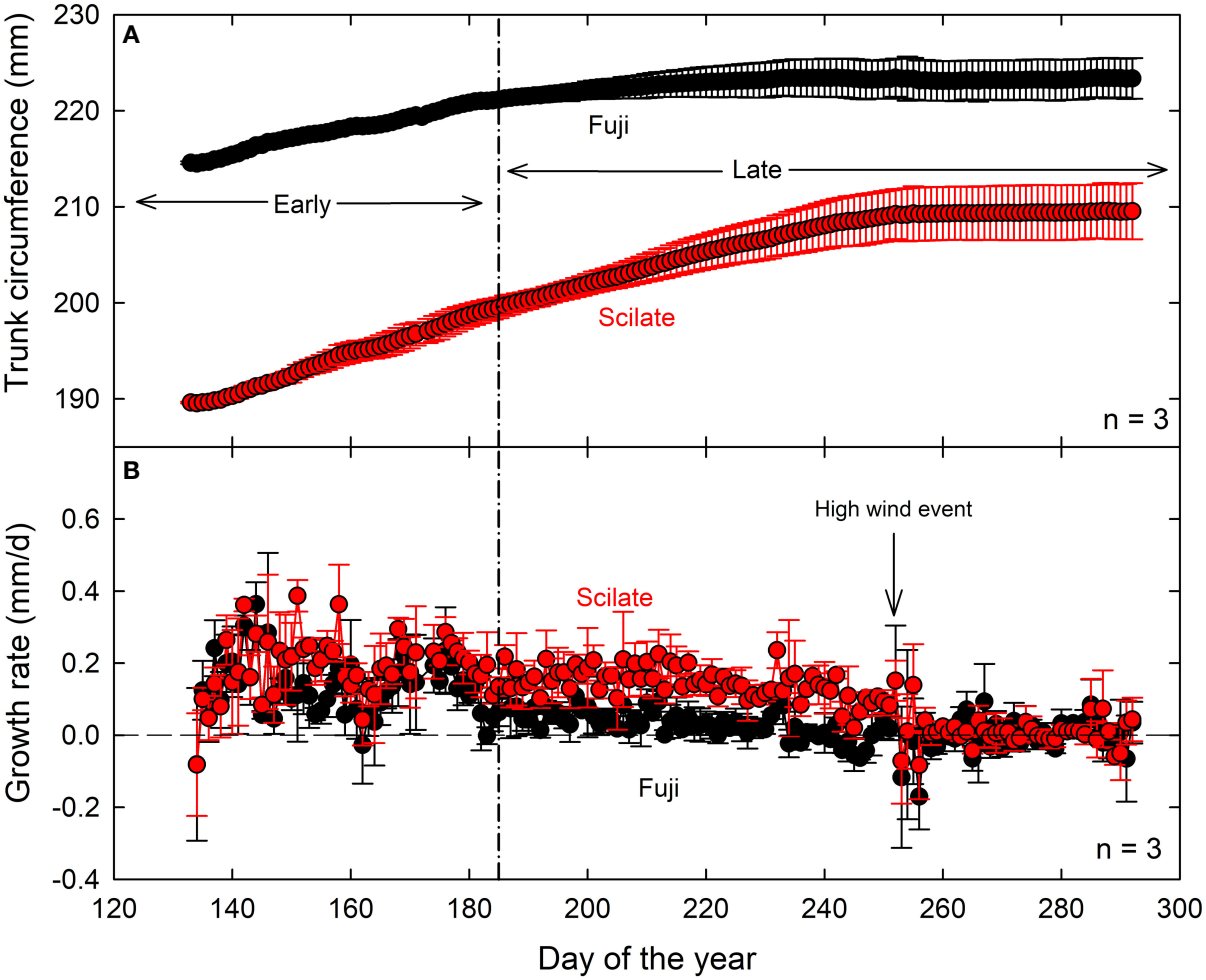
Maximum daily circumference **(A)** and daily circumferential growth rate **(B)** measured by band dendrometers of three replicate trees from five-year-old ‘Aztec Fuji’ and ‘Scilate’ (Envy™) fruiting scions on Malling 9 rootstocks over the course of the growing season. Data are normalized to average ending circumference, measured by hand, for each scion to show growth patterns. A severe weather event occurred on day 252 of the year in which gusts of wind reached up to 40 m/s caused severe damage to the orchard.

Starting average circumferences were significantly different between Fuji and Scilate, with Fuji averaging 21.4 ± 0.02 cm and Scilate averaging 19.0 ± 0.09 cm (P = 0.01). This significant difference (P = 0.03) persisted at the end of the trial period with final trunk circumference of Fuji averaging 22.3 ± 0.7 cm and Scilate averaging 21.0 ± 0.8 cm (**Table 1**). Total circumferential growth was greater in Scilate (P< 0.01), with circumferences increasing by an average of 2.0 ± 0.4 cm while Fuji circumferences increased by an average of 0.9 ± 0.3 cm over the trial period (**Figure 3**). Differences in the pattern of circumferential growth were also noted, with Fuji rapidly putting on growth early in the season and then plateauing later season, while Scilate steadily put on growth until late in the season.

**Table 1:** Fruit harvest parameters and final trunk and stem measurements for ‘Aztec Fuji’ and ‘Scilate’ (Envy™) scions grafted to Malling 9 Selection NIC™ 29 rootstocks.

	Harvest Weight	Crop load	Crop load/TCSA	Fruit Size	Ending circumference	Trunk cross sectional area (TCSA)	Shoot elongation
Scion	kg fruit/tree	#/tree	#/cm^2^ TCSA	g/fruit	cm	cm^2^	cm
Fuji	22.2	119	3.08	193	22.3	40.1	33.7
Scilate	11.7	52	1.49	228	21	35.3	31
p value	< 0.01	< 0.01	< 0.01	< 0.01	0.03	0.03	0.07

A severe weather event occurred on day of the year 252 in which gusts of wind reached up to 40** m/s** causing a significant amount of fruit loss (~50%).

Daily TGR was significantly different (P = 0.02) between the two cultivars over the course of the trial, with Scilate cultivars averaging higher TGR. Analysis of TGR did not find strong correlations to atmospheric drivers R_n_ (Fuji, r² = 0.05; Scilate, r² = 0.23), T_a_ (Fuji, NS; Scilate, r² = 0.13), VPD (Fuji, r² = 0.03; Scilate, r² = 0.08) or ET_r_ (Fuji, NS; Scilate, r² = 0.30) (**S1 Figure**). Daily TGR was also not significantly correlated to θ at the 20 cm level and poorly correlated at the 80 cm level (Fuji, r² = 0.28; Scilate, r² = 0.12). Poor or not significant correlations of TGR to Ψ_stem_ were observed (Fuji, r² = 0.10; Scilate, NS) (**S1 Figure**).

Measurements of MDS were not significantly different over the trial period (P = 0.30). Data were still segregated by scion for analysis because of differences in crop load and ending circumferences. Linear regression analysis was used to examine correlations to environmental variables while controlling for phenological stage (before and after DOY 185) (**Table 2**).

**Table 2:** Coefficients of determination.

Parameter	Season	Cultivar	R_n_	T_a_	VPD	ETr	θ at 20 cm	θ at 80 cm
			(MJ m^-2^ d^-1^)	(C)	(VPD)	(mm day^-1^)	(cm^3^ cm^-3^)	(cm^3^ cm^-3^)
MDS	Early	Fuji	0.2	0.61	0.53	0.46	0.17	0.3
		Scilate	0.15	0.64	0.56	0.45	0.19	0.22
	Late	Fuji	0.71	0.45	0.45	0.69	0.39	NS
		Scilate	0.68	0.61	0.61	0.75	0.3	0.08
SFI	Early	Fuji	0.37	0.84	0.77	0.76	NS	0.04
		Scilate	0.33	0.88	0.87	0.83	NS	NS
	Late	Fuji	0.74	0.79	0.78	0.88	0.12	NS
		Scilate	0.73	0.8	0.82	0.89	0.11	NS
Ψ_Stem_	Early	Fuji	0.22	0.7	0.76	0.7	0.12	0.12
		Scilate	0.19	0.67	0.77	0.64	0.03	0.04
	Late	Fuji	0.39	0.42	0.31	0.46	0.59	0.23
		Scilate	0.37	0.46	0.4	0.45	0.42	0.25

Coefficients of determination (r²) between environmental inputs and maximum daily trunk shrinkage (MDS), sap flow index (SFI), and mid-day stem water potential (Ψ_Stem_) for ‘Aztec Fuji’ and ‘Scilate’ (Envy™) scions grafted to Malling 9 Selection NIC™ 29 rootstocks. Both scion/rootstock combinations were subjected to multiple dry down events over the course of the trial. NS indicates no statistically significant regression.

Early in the season, MDS of both Fuji and Scilate were most highly correlated to T_a_ (Fuji, r² = 0.61; Scilate r² = 0.64), followed by VPD (Fuji, r² = 0.53; Scilate r² = 0.56), and ET_r_ (Fuji, r² = 0.46; Scilate r² = 0.45). Later in the season, scion correlation patterns to environmental drivers differed, with Fuji being most correlated to R_n_ (r² = 0.71), followed by ET_r_ (r² = 0.69) and then T_a_ (r² = 0.45) and VPD (r² = 0.45). In the Scilate scions the two most significant correlations were flipped compared to Fuji, with ET_r_ (r² = 0.75) being the most highly correlated followed by R_n_ (r² = 0.68), and T_a_ (r² = 0.45) and VPD (r² = 0.45) being equal. Maximum daily shrinkage was less correlated to θ than atmospheric variables. Early in the season θ at 80 cm depth was more correlated to MDS (Fuji r² = 0.30; Scilate r² = 0.22) than θ at 20 cm (Fuji r² = 0.17; Scilate r² = 0.19). This pattern flipped later in the season with θ at 20 cm being better correlated to MDS (Fuji r² = 0.39; Scilate r² = 0.30) than θ at 80 cm (Fuji NS; Scilate r² = 0.08). When pooling MDS data by scion and across early and late season, linear regression analysis identified ET_r_ as the most significant atmospheric determinant of MDS and θ at 20 cm the most significant soil moisture depth. Multiple linear regression model utilizing ET_r_ and θ at 20 cm accounted for 77% of variation in MDS.

### 3.3 Stem water potential

**Figure 4:**
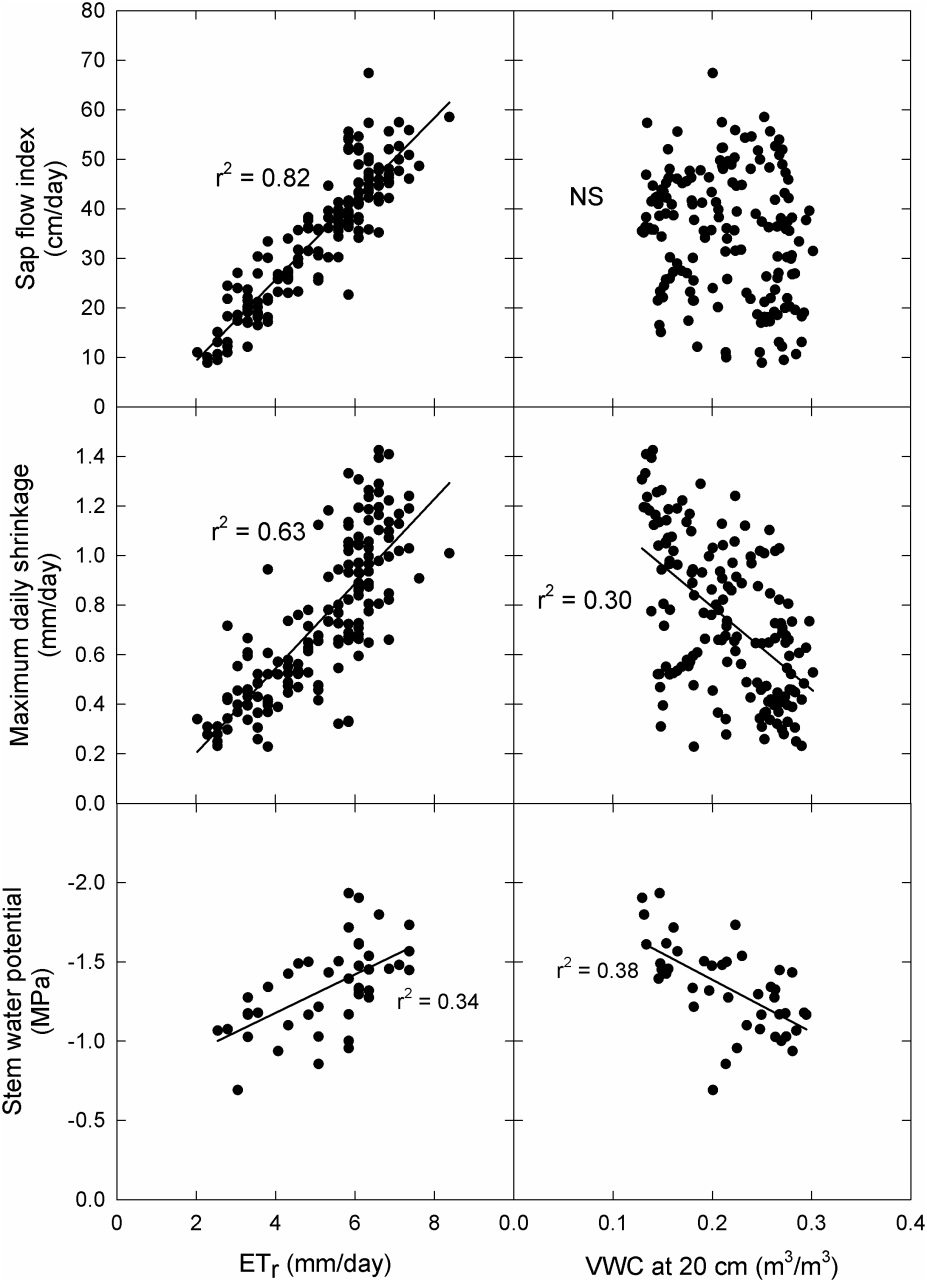
Correlations to principal above and below ground environmental drivers across scion type. Correlations between maximum daily trunk shrinkage, sap flow index, and mid-day Ψ_stem_ and ETr and volumetric water content at 20 cm. Data were pooled for ‘Aztec Fuji’ and ‘Scilate’ (Envy™) scions on Malling 9 rootstocks and over the entire season. NS indicates no statistically significant regression.

There was no significant difference in Ψ_Stem_ (P = 0.93) between the Fuji and Scilate scions with Fuji averaging -1.39 ± 0.07 MPa and Scilate averaging -1.23 ± 0.08 MPa over the course of the growing season (**Figure 2**). Scions were separated for analysis based on differences in harvest and final growth data. Responses to all environmental variables were examined using linear regressions (**Table 2**). Early season responses of Ψ_Stem_ in both Fuji and Scilate showed strong correlations to VPD (Fuji, r² = 0.76; Scilate, r² = 0.77), T_a_ (Fuji, r² = 0.70; Scilate, r² = 0.67) and ET_r_ (Fuji, r² = 0.70; Scilate, r² = 0.64). During the late season Ψ_Stem_ responses showed medium correlations to almost all measured environmental variables, apart from wind speed. For Fuji scions the most significant correlations in declining order were θ at 20 cm (r² = 0.59), ET_r_ (r² = 0.46), T_a_ (r² = 0.42), R_n_ (r² = 0.39), VPD (r² = 0.31), and θ at 80 cm (r² = 0.25). In Scilate scions most significant correlations were: T_a_ (r² = 0.46), ET_r_ (r² = 0.45), θ at 20 cm (r² = 0.42), VPD (r² = 0.40), R_n_ (r² = 0.37), and θ at 80 cm (r² = 0.25). When pooling Ψ_Stem_ data across scion and phenological stage, multiple linear regression modeling utilizing ET_r_ and θ at 20 cm accounted for 41% of variation in Ψ_Stem_. Pooled scion Ψ_Stem_ was also examined for correlations to pooled MDS and SFI (**Figure 4**). Controlling for seasonality improved correlation coefficients between SFI and MDS. Early season data were more correlated with MDS measurements (r² = 0.85) than SFI (r² = 0.69). Late season correlations declined in both MDS and SFI, though MDS remained well correlated (r² = 0.71), while SFI correlations declined significantly (r² = 0.36).

### 3.4 Sap flow index

Sap flow index was not significantly different (p = 0.97) between the two grafted scions over the course of the season (**Figure 2**). Early in the season, both scions were highly correlated to T_a_ (Fuji, r² = 0.84; Scilate, r² = 0.88), VPD (Fuji, r² = 0.77; Scilate, r² = 0.87), and ET_r_ (Fuji, r² = 0.76; Scilate, r² = 0.83) (**Table 2**). Later in the season this trend remained the same with correlations to ET_r_ improving (Fuji, r² = 0.88; Scilate, r² = 0.89) followed by T_a_ (Fuji, r² = 0.79; Scilate, r² = 0.80), VPD (Fuji, r² = 0.78; Scilate, r² = 0.82), and R_n_ (Fuji, r² = 0.74; Scilate, r² = 0.73). There were no significant correlations to wind speed or θ at either the 20 or 80 depth during either the early or late season. When pooling data by scion and season, linear regression analysis found that ET_r_ (r² = 0.82) was the most significant atmospheric driver and θ at 20 and 80 cm were not significantly correlated (**Figure 4**)(**S2 Figure**). Multiple linear regression modeling using ET_r_ and θ at 20 cm accounted for 84% of variation in SFI readings.

### 3.5 Measurement variability

Stem water potential had a combined season long coefficient of variation ( $CV=\frac{Standard\;deviation}{Mean}$ ) of 12.4% with average CV values being slightly lower for Fuji (9.5%) than Scilate (11.2%) (**Table 3**). Maximum daily shrinkage had a combined CV of 31.9% with Fuji being slightly more variable (29.3%) than Scilate (25.8%). Sap flow indexes were the most variable, having an overall CV of 37.1% with Fuji being significantly (47.0%) more variable than Scilate (25.7%).

**Table 3:** Variability of measurements.

Scion	Ψ_Stem_	MDS	SFI
Fuji	9.50%	29.30%	47.00%
Scilate	11.20%	25.80%	25.70%
Combined	12.40%	31.90%	37.10%

Coefficients of variation (%) of midday stem water potential (Ψ_Stem_), maximum daily shrinkage (MDS) and sap flow index (SFI) measured on fruiting ‘Aztec Fuji’ and ‘Scilate’ (Envy™) both grafted to Malling 9 Selection NIC™ 29 rootstocks. Values are presented for data over the course of the season from four replicate trees for measures of SFI and Ψ_Stem_ and three replicate trees for MDS. SFI and MDS are daily averages for a 151 day trial period while Ψ_Stem_ average 43 different measurements over the course of the season.

### 3.6 Harvest and final growth

‘Aztec Fuji’ scions had significantly higher (P > 0.01) fruit harvest mass per tree averaging 22.2 ± 4.0 kg per tree while the Scilate averaged 11.7 ± 1.8 kg per tree (**Table 1**). Individual fruit mass was higher (P = 0.02) in Scilate averaging 227.6 ± 9.2 g per fruit compared to Fuji which average 192.9 ± 10.3 g per fruit. ‘Aztec Fuji’ had more (P > 0.01) fruit per tree, averaging 119 ± 15 compared to Scilate which averaged 52 ± 4 fruit per tree. Normalizing for trunk cross sectional area (TCSA) Fuji maintained higher numbers of fruit averaging 3.1 ± 0.6 fruits per cm² of TCSA while Scilate averaged 1.5 ± 0.2 fruits per cm² of TCSA. Stem elongation in Fuji appeared slightly more (33.7 ± 0.6 cm) than Scilate (31.0 ± 1.4 cm), but differences were not significant (p = 0.07).

## 4 Discussion

### 4.1 Trunk circumferential variation and growth

Studies have shown that the phenological stages of growth in orchard trees can impact the response of MDS to environmental drivers and Ψ_Stem_ (Egea et al., 2009; Marsal et al., 2015). In particular, Liu et al. (2012) identified two seasonal stages of growth in apple (cv. Golden Delicious) based on trunk diameter growth and leaf area index. During the first stage, which was characterized by rapid leaf area and trunk expansion, trees emerged from dormancy and anthesis occurred. The second stage was marked by a plateauing of trunk growth and leaf area index, coupled with rapid expansion and maturing of fruit. In this study, we observed a plateauing of maximum daily trunk circumference in the Fuji scion around day 185 (3 July), although it was less pronounced than reported by Liu et al. (2012) (**Figure 2**). On the other hand, we noticed only minor slowing of growth in the Scilate cultivar, with circumference measurements continuing to increase until late in the growing season. While fruit size was larger in the Scilate scions, overall crop load (fruit #/cm^2^ TCSA) was lower (**Table 1**). Shoot elongation was not statistically different at α of 0.05, which, given smaller TCSA and lower crop load in the Scilate, suggests greater carbon allocation to vegetative growth. Furthermore, continued circumferential growth later in the season in Scilate scions was likely due to lighter crop load and reduced carbon allocation to fruiting compared to Fuji.

After controlling for phenological stage of growth, it was observed that the correlations of MDS to Ψ_Stem_ were strongest early in the season and declined after DOY 185 (July 3^rd^) when fruit development became the dominant factor affecting tree responses. This finding was consistent with previous studies using point dendrometers that have documented declining correlations of MDS to Ψ_Stem_ in plum and peach as the season progressed (Intrigliolo and Castel, 2006; Marsal et al., 2015). In this study, reduced correlations of MDS to Ψ_Stem_ in the late season are attributed to a combination of factors, including fruit load and extended drought.

**Figure 5:**
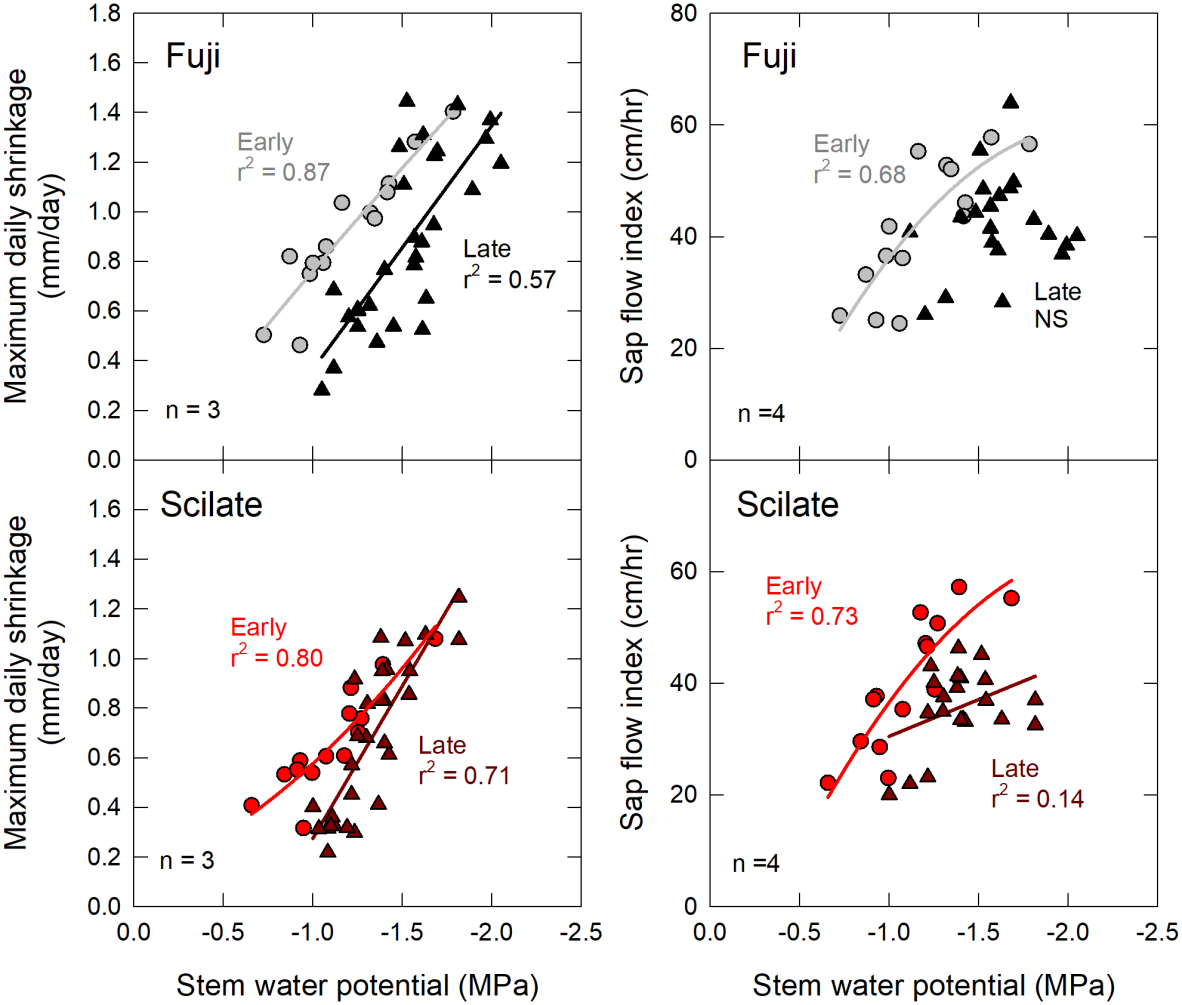
Early and late season correlations to stem water potential. Correlations of maximum daily trunk shrinkage and sap flow index to mid-day Ψ_stem_ for fruiting scions ‘Aztec Fuji’ and ‘Scilate’ (Envy™) on Malling 9 rootstocks. Data were segregated into early (light grey and red circles) season responses that corresponded with spur leaf development and fruit set, and late season (black and dark red triangles) responses which corresponded with elongation of terminal and bourse shoots. NS indicates no statistically significant regression.

The onset of fruit development increases osmotic loading of the phloem, which impacts water storage dynamics of the tree (Ortuño et al., 2010). Under well-watered conditions, osmotic loading can result in greater swelling of cambium tissues during nighttime recharge of water (Wang et al., 1995), resulting in increased MDS readings in relation to Ψ_Stem_ (Intrigliolo and Castel, 2007). However, in this study, MDS values decreased for the same Ψ_Stem_ (**Figure 5**), indicating that the opposite trend was observed. Moreover, although the Fuji had higher yield and crop load at harvest, MDS values were not significantly higher (p = 0.30) than those of the Scilate (**Table 1**). Soil moisture content and Ψ_Stem_ were on average lower throughout the plot after DOY 185 indicating greater water stress during fruiting. This limited soil water availability could have inhibited nighttime recharge of stem water and diurnal trunk expansion. Additionally, stored water can account for up to 50% of transpirational demand (Köcher et al., 2013). More negative osmotic potential in the cambium tissues could have acted as a competitive sink for stored water, limiting trunk contraction due to transpirational loss. The combination of greater osmotic loading and limited soil water availability could in this way depress MDS responses.

Previous studies have suggested that MDS values follow a parabolic response to Ψ_Stem_, with MDS values increasing until a species-specific threshold is reached, after which they decline (Ortuño et al., 2010). This response has been attributed to various factors, including depletion of water reservoirs in the phloem and surrounding xylem tissues as well as stomatal and osmotic regulation (Garnier and Berger, 1986; Remorini and Massai, 2003). However, in this study, MDS values increased linearly with more negative Ψ_Stem_. Ortuño et al. (2010) reported MDS began to decline after Ψ_Stem_ values reached -2.5 MPa in potted apple trees. Minimum Ψ_Stem_ values in this study reached ~ -2.0 MPa at which time leaf curling, tip burn and low θ were observed. This was judged to be significant water stress, beyond what would be tolerated in commercial production.

Although previous research has suggested that daily growth is a better indicator of drought stress than MDS in rapidly growing young trees (Nortes et al., 2005), significant correlations between daily growth and environmental parameters or Ψ_Stem_ were not observed in this study. Trees were at the 6^th^ leaf at the time of the study, which is considered to be mature and past the stage of initial rapid growth in which daily growth is the most sensitive parameter for water stress.

### 4.2 Sap flow index

Previous research has indicated that the correlation between sap flow and environmental drivers can vary depending on the phenological stage of the tree (Chen et al., 2014; Tie et al., 2017). For instance, daily sap flow rates presented by Liu et al. (2012) showed that correlations of sap flow to T_a_, VPD, and ET_r_ improved over the course of the season. Similarly, data presented in this study found that correlations of R_n_ and ET_r_ with sap flow increased over the season. However, correlations to T_a_ and VPD showed no significant improvement (**Table 2**). Liu et al.’s (Liu et al., 2012) findings could be attributed to the inclusion of data from early spring when trees had fully leafed out, resulting in low sap flow readings and thus low correlations. Tie et al. (2017) addressed this issue by normalizing the data to leaf area index, leading to improved correlation coefficients throughout the season. In this study, data collection was initiated after trees had leafed out, which improved early season correlations and more closely matched data from Tie et al. (2017). Previous studies have identified VPD, R_a_, T_a_, θ, and leaf area index as predominant drivers of sap flow in various trees and environmental conditions (Ford et al., 2004; Dragoni et al., 2005; Agam et al., 2013; Mobe et al., 2020). In this study ET_r_, VPD, and T_a_ were most strongly correlated to SFI, while R_a_ was only well correlated later in the season. Interestingly, θ was not significantly correlated to SFI at any point in time. Shifts in seasonal SFI response were most likely due to older leaves accounting for a larger portion of the tree canopy, which are less responsive to environmental drivers due to greater internal shading and reduced stomatal conductance compared to recently matured leaves (Constable and Rawson, 1980; Flore and Lakso, 2011).

Correlations to ET_r_ were high during both the early and late season and showed significant overlap, and seasonal correlations pooled across scion type were also strong (**Figure 4**). High correlations of SFI to ET_r_ was unexpected given the published literature which points to divergence in orchard ET from reference ET (Jarvis, 1984; Dragoni et al., 2005). Reference ET models utilize idealized values for canopy characteristics that reflect a continuous, short, dense, and homogenous crop with a relatively large boundary layer. Because of these assumed canopy characteristics and boundary layer effects, reference ET models have been better correlated to incoming solar radiation than bulk atmospheric conditions (Jarvis, 1984). Tall, widely spaced trees coupled with the large amount of self-shading mean that bulk atmospheric conditions (e.g. VPD) is often a better predictor of orchard ET values (Dragoni et al., 2005). We hypothesize that in high density orchard plantings with interconnected canopies, aerodynamic resistance is higher creating boundary layer effects that decouple orchard ET from bulk air properties, especially later in the season as leaf area index reaches a maximum. Additionally, highly managed fruit tree orchards utilize pruning and training techniques to maximize canopy radiation capture.

Previous research has shown linear correlations of sap flow readings to Ψ_Stem_ under non-limiting soil water conditions (Ortuño et al., 2010). Both early and late seasonal responses followed this same pattern of increasing sap flow with decreasing Ψ_Stem_ (**Figure 5**). Correlations of SFI to Ψ_Stem_ were stronger in the early season while late season correlations decreased. Declining correlations later in the season could be a function of stomatal regulation, however fruiting deciduous orchards have been shown to have higher stomatal conductance, transpiration and carbon assimilation than de-fruited trees of the same age (Naor et al., 2008). Similar to depressed correlations of MDS, SFI correlations to Ψ_Stem_ most likely declined due to limited soil water availability combined with solute loading, leaf age and crop load.

Several studies have reported reductions of peak sap velocities following severe drought. Researchers have also noted an inward radial shift of peak velocities toward the heartwood (Cermak and Nadezhdina, 1998; Ford et al., 2004). Based on these findings Nadezhdina et al. (2007) suggested that analysis of the shape of the sap wood profile might be a reliable indicator for irrigation scheduling. Studies in olive, apple and Asian pear however did not find significant variation in sap velocity profiles under a range of soil water availability and atmospheric demand (Fernández et al., 2008). Our analysis of the ratio of outer to inner SFI found a moderate correlation to Ψ_Stem_ when pooling scions (r² = 0.48) (**S3 Figure**). It may be possible that the proposed technique is not applicable for diffuse porous species such as apple, but could work for ring porous species which have a bimodal radial distribution of vessel diameters. This means that they do not produce steep gradients in sap velocity between the inner and outer sapwood (Bush et al., 2010; Tyree and Zimmermann, 2013).

### 4.3 Stem water potential

A saturating response of season long Ψ_Stem_ to VPD has previously been reported in apple (De Swaef et al., 2009) where a linear relationship has been described in olive (Moriana et al., 2012), prune (Fereres and Goldhamer, 2003) and plum (Intrigliolo and Castel, 2006). De Swaef et al. (2009) (De Swaef et al., 2009) speculated that the saturating response seen in apples may be due to restricted root volumes. In dwarfing rootstocks, like the one used in this study, lower rootzone volumes have been associated with reduced drought tolerance (Tworkoski et al., 2016). When Ψ_Stem_ is pooled across scions and seasonally a polynomial function produces a greater fit (r² = 0.62; **S4 Figure**) than a simple linear one (r² = 0.53; **S4 Figure**) making data presented here in line with observation by De Swaef et al. (2009). Overall Ψ_Stem_ was less impacted by environmental drivers then SFI and MDS with division of readings by seasonality improving early season correlations and depressing correlations later in the season. It is unclear from this study if phenological stage of growth affects Ψ_Stem_ response or if drier conditions later in the season caused seasonal differences in response.

### 4.4 Discussion of relative sap flow index

The accurate calibration of absolute sap flow using heat pulse techniques involves several technical calibrations, including measuring sapwood density, sapwood moisture content, area of conducting tissue, correcting needle misalignment, and determining the thermal diffusivity of the sapwood (Taylor et al., 2013). However, these complex calibrations present significant challenges to the adoption of sap flow as an irrigation tool in commercial orchards where equipment and expertise may not be available. Even in research settings, accurate calibration for absolute sap flow is difficult, with an average error rate of 34% in published studies and most measurements underestimating tree water usage (Forster, 2017).

Determinations of sap flow rely on measured changes in temperature and time elapsed to calculate the velocity of a pulse of heat as it is carried through the trunk (Swanson and Whitfield, 1981; Cohen, 1991; Burgess et al., 2001). Thermal accounting of conductive and convective properties of the trunk and sap are then employed to derive sap velocity from heat velocity and estimates of conducting tissue area are subsequently used to estimate sap flow. Typically, thermal properties are determined through either coring the tree before the trial or destructively harvesting the tree after the trial, and these properties are then treated as constants throughout the measurement campaign. In this study we focused solely on measurements of heat velocity, without attempting to derive accurate measures of sap flow. By simplifying the application and removing the need for technical calibrations, we remove barriers to use. The use of relative measures of sap flow is not without precedent, several researchers have used either indexes (Nadezhdina, 1999), normalized values (Burgess and Dawson, 2007; Ballester et al., 2012) or relative measures (Doronila and Forster, 2015) of sap flow in tree water use analysis. We argue that understanding the underlying pattern of sap flow is essential in informing tree responses to water stress, and absolute measurements are not necessary for this purpose. The SFI values presented here follow trends seen in the literature of calibrated sap flow, as described above.

### 4.5 Measurement variability

High tree-to-tree measurement variability has been cited as a reason for limited adoption of sap flow sensors and dendrometers as irrigation tools in commercial orchards (Ortuño et al., 2010; Fernández, 2017). In analyzing coefficients of variation ( $CV=\frac{Standard\;deviation}{Mean}$ )for this trial we found that MDS and SFI were more variable than Ψ_Stem_ (**Table 3**). Naor and Cohen (2003) (Naor and Cohen, 2003) noted the same pattern in apple with Ψ_Stem_ having the least tree to tree variability, followed by MDS and SFI. The study authors attributed high variability of MDS to variability in vasculature area and tree hydraulic conductance. Variability in SFI was speculated by the authors to be due to tree-to-tree differences in canopy size and thus rates of transpiration. Plant water status on the other hand is a more holistic measure of plant response that incorporates many different crop characteristics and physiological responses (Naor et al., 2005). The higher degree of variability in MDS and SFI is also partially explained by the high degree of environmental correlation of these measures. When pooling data by scion and season, multiple linear regression utilizing ET_r_ and θ at 20 cm accounted for 41% of reading variation of Ψ_Stem_ compared to 77% of the reading variation in MDS, and 85% of SFI. Previous studies have compared the signal intensity (SI) of MDS, sap flow and Ψ_Stem_ by contrasting readings from well-watered trees to those of deficit irrigated or drought stressed trees. While utilizing SI reduced the amount of environmental variability, physiological parameters followed the same pattern seen in this study with Ψ_Stem_ having the lowest variability, followed by MDS and SFI (Fernández and Cuevas, 2010). Overall CV of MDS from band dendrometers used in this study was 31.9% which is higher than studies CV values for point dendrometers summarized by Fernández and Cuevas (2010), which averaged 12.5% for fruit trees. This result was surprising given that circumferential measurements incorporate a larger area and reduce viability. Greater variability necessitates more instrumentation to reduce sampling errors, this in turn drives up the initial investment cost of any sensor-controlled irrigation system. These costs however, should be measured against the amount of labor and time needed to collect Ψ_Stem_ measurements and the ability of automated measurements to more comprehensively capture seasonal records.

### 4.6 Irrigation scheduling and future considerations

Previous researchers have automated irrigation based on set thresholds of MDS (Bussi et al., 1999) or sap flow (Cohen, 1991). Some researchers have sought to overcome the impact of environmental drivers and phenological stages on MDS and sap flow by using ratios of automated irrigation treatments to well-watered controls (Ortuño et al., 2006). Other studies have relied on mechanistic models based on sap flow and trunk water storage have been used to estimate Ψ_Stem_ and automate irrigation (Steppe et al., 2008). However, the long-term adoption and large-scale application of these strategies are hindered by the issue of sensor variability and the technical expertise required for implementation. This study primarily focused on sensor responses in high density apple and the evaluation of alternative sensors and sensor readings. Although more easily adopted for commercial application, the variability of these alternative sensors and sensor readings was similar or greater than that reported in the literature. Future research should focus on easily adopted sensor readings that can reliably distinguish physiological responses related to drought from responses due to simple environmental variation and changes in phenological stage.

In addition to addressing sensor responses, sensor-based irrigation scheduling must also consider how signals can inform irrigation application amounts. Steppe et al. (2008) based irrigation amounts on measured sap flow, while other researchers have used trunk diameter and sap flow trigger a timed irrigation application. One limitation of using a sap flow index as suggested in this study is it does not provide an absolute measure of sap flow and thereby an estimation of tree water usage. However, a responsive enough signal of plant water stress that can directly trigger on demand irrigation could bypass the need to calculate specific irrigation amounts (Jones, 2004). Furthermore, tree-derived estimates of irrigation amount must take into account not only tree water uptake but also percolation, competitive uptake from surrounding crops, evaporative loss and ground water recharge from below. When considering water use from instrumented crops alone, this may lead to underestimation irrigation amounts that need to be compensated.

Finally, irrigation application must consider its effects on yield and growth which are the ultimate goals in irrigation management. Previous research focusing on regulated deficit irrigation has shown controlled drought through the withholding irrigation applications to be effective in a number of fruit tree species. This practice has the additional documented benefits of: reduced vegetative growth and reductions in labor hours associated with pruning, improved postharvest quality and fruit shelf life, and greater resilience to pathogens (Goldhamer et al., 2005). Much research has focused on linking physiological measurements determined through automated data acquisition to Ψ_Stem_, for which there is a large body of reported literature linking values to yield and fruit size. A mechanistic approach could consider photosynthetic rate and carbon partitioning of the tree and develop a predictive model that would incorporate automated irrigation amounts and timing to control fruit development (Steppe et al., 2008). However, implementing such an approach would again require technical expertise to implement, posing a barrier for adoption.

## 5 Conclusions

Given the difficulty of calibrating heat pulse sensors to derive absolute sap flow, relative values provide a reliable indication of sap flow responses to environmental conditions while lowering barriers to entry. However, even relative sap flow is not a reliable method for irrigation scheduling and sap flow sensors must be replaced each year.

Band dendrometers provide significant advantages over point dendrometers because they can be quickly and non-invasively installed or removed. Maximum daily shrinkage (MDS) measurements were well correlated to Ψ_Stem_. In semiarid regions, MDS measurements could replace labor intensive Ψ_Stem_ measurements.

Controlling for seasonality improved correlations of MDS and SFI to Ψ_Stem_. Correlations declined as the season progressed, likely due to crop loading, soil water availability and leaf age. SFI, however, was highly correlated with ET_r_ throughout the season. Our results suggest that high density plantings create a more continuous surface for aerodynamic resistance so that ET_r_ is more highly correlated than in widely-spaced trees in traditional orchards. Thus, orchard density needs to be considered when scheduling irrigation.

This study highlights the complex interplay between environmental conditions and tree water use dynamics, which can vary depending on species and phenological stage. MDS and sap flow measurements can provide insights into tree water status, but it is important to consider fruit load and soil moisture content when interpreting these data.

## Data availability statement

The original contributions presented in the study are included in the article/**Supplementary Material**. Further inquiries can be directed to the corresponding author.

## Author contributions

WW, BBu, and BBl contributed to the design of the study, statistical analysis and interpretation of the data. WW wrote the first draft of the manuscript. BBu and BBl provided revisions to the manuscript and figures. All authors contributed to the article and approved the submitted version.
