# Supplementary material for: Assessing water stress in a high-density apple orchard using trunk circumference variation, sap flow index and stem water potential
Source: Front Plant Sci. 2023 Aug 3;14:1214429. doi: 10.3389/fpls.2023.1214429 (PMC10435262; doi:10.3389/fpls.2023.1214429)
Supplement: Supplementary file 1 [file DataSheet_1.docx]

Supplementary Material

Assessing water stress in a high-density apple orchard using trunk circumference variation, sap flow index and stem water potential

William D. Wheeler^*^, Brent Black, Bruce Bugbee

*** Correspondence:** William Wheeler: william.wheeler@agnet.tamu.edu

## Supplementary Figures


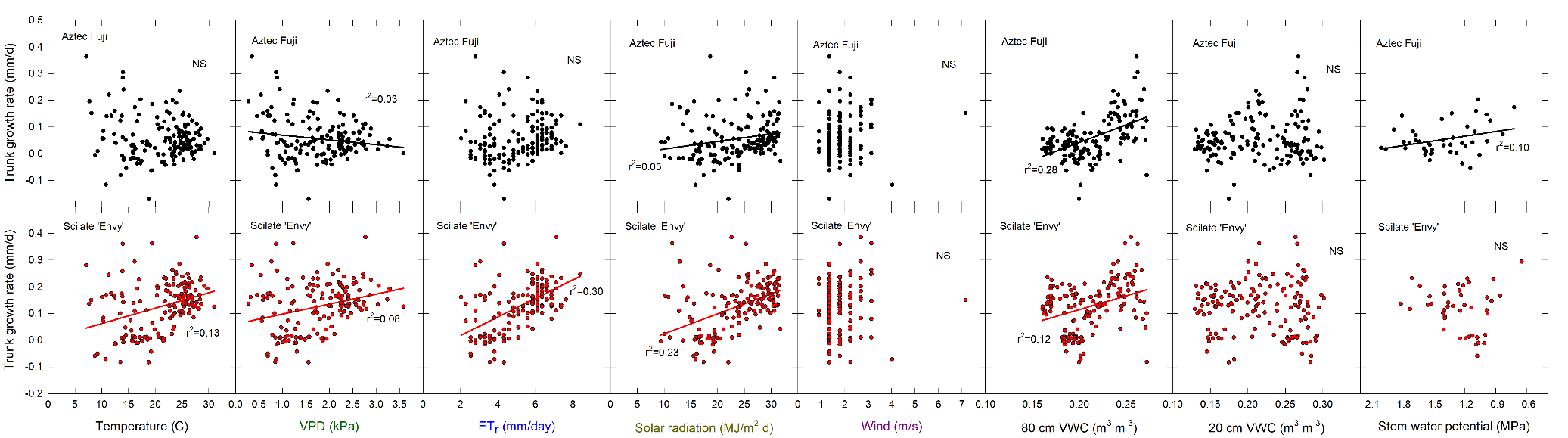


Supplementary Figure 1. Trunk growth rate correlations to environmental measurements for ‘Aztec Fuji’ and ‘Scilate (Envy™)’ fruiting scions grafted to Malling 9 dwarfing root stocks. Daily average measurements separated by scion type across the 2020 grown season. Error bars omitted for clarity, n=4. NS indicates no statistically significant correlation.


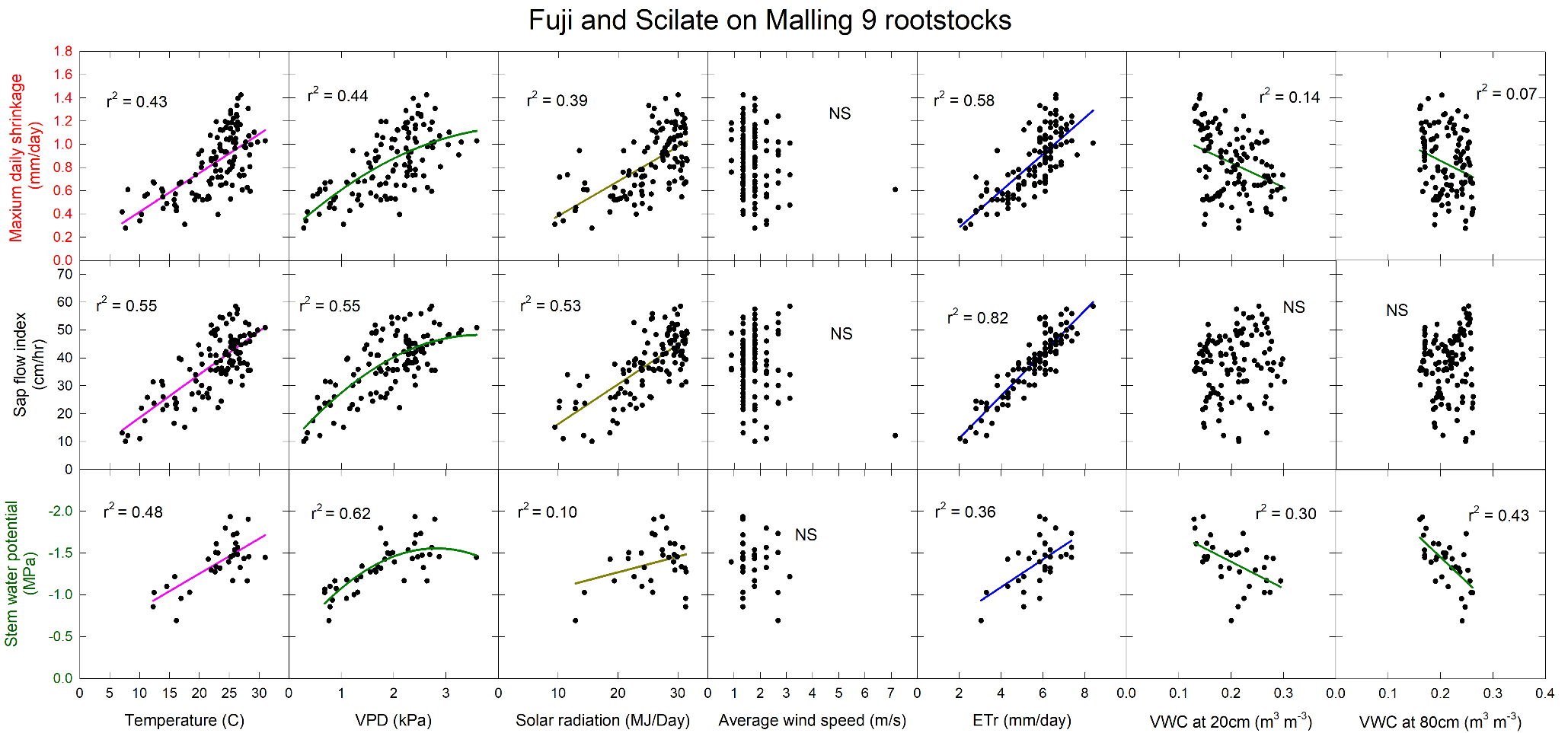


Supplementary Figure 2. Maximum daily shrinkage, sap flow index, and stem water potential correlations to environmental measurements across two scions of high density apple. Combined correlations of daily measurements from fruiting scions ‘Aztec Fuji’ and ‘Scilate’ (Envy™) on Malling 9 rootstocks. Correlations were modeled using linear regression with the exception of vapor pressure deficit (VPD) which used a polynomial fit based on saturating responses of physiological measurements. Error bars omitted for clarity, n=8. NS indicates no statistically significant correlation.


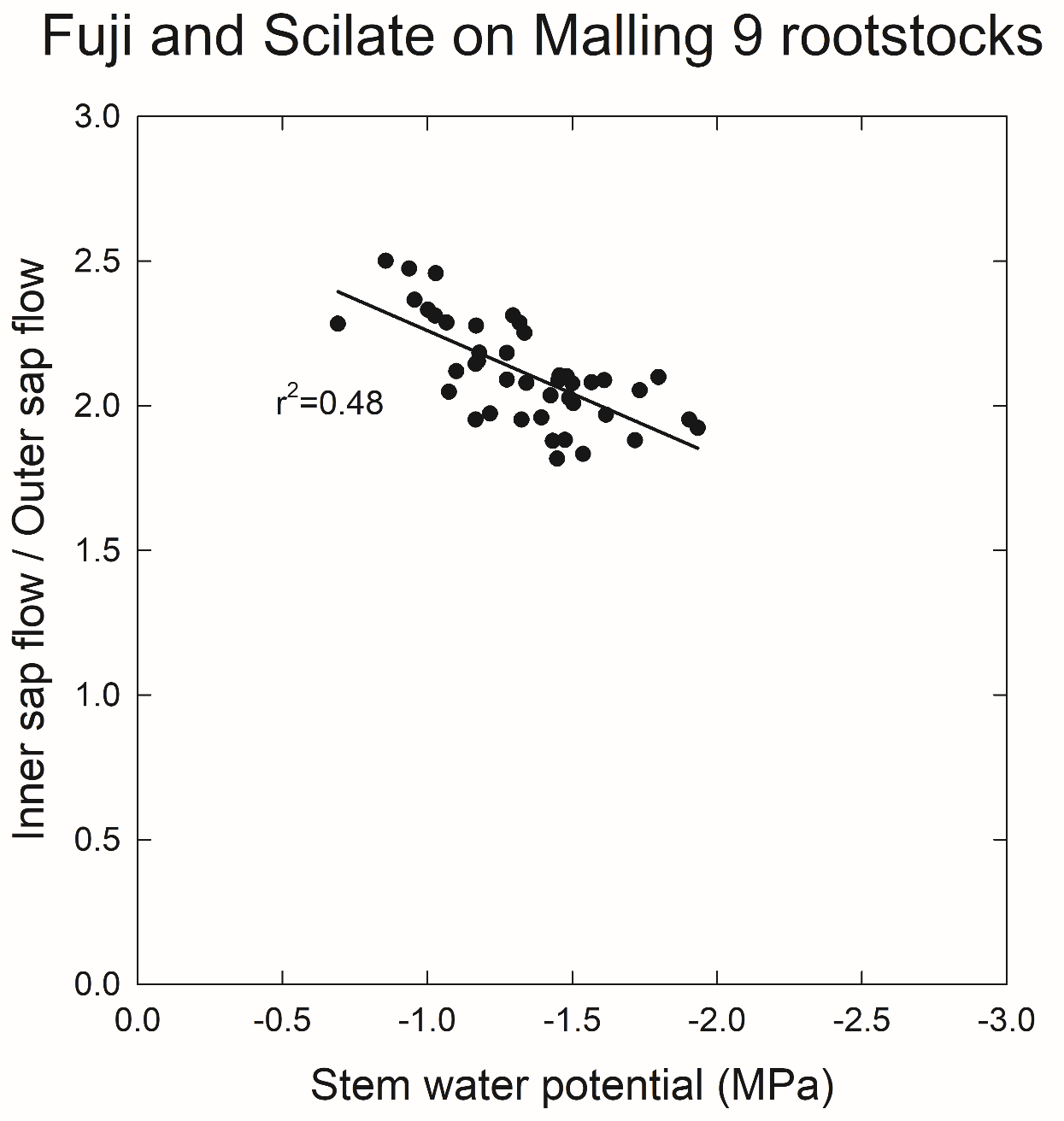


Supplementary Figure 3. Correlation of the ratio of inner sap flow and outer sap flow to stem water potential. Daily averages of inner/outer sap flow and stem water potential from fruiting scions ‘Aztec Fuji’ and ‘Scilate’ (Envy™) on Malling 9 rootstocks. Previous research has noted an inward shift of peak sap velocities toward the heart wood under drought conditions and suggested that this may be a reliable indicator for irrigation scheduling. Error bars omitted for clarity, n=8.


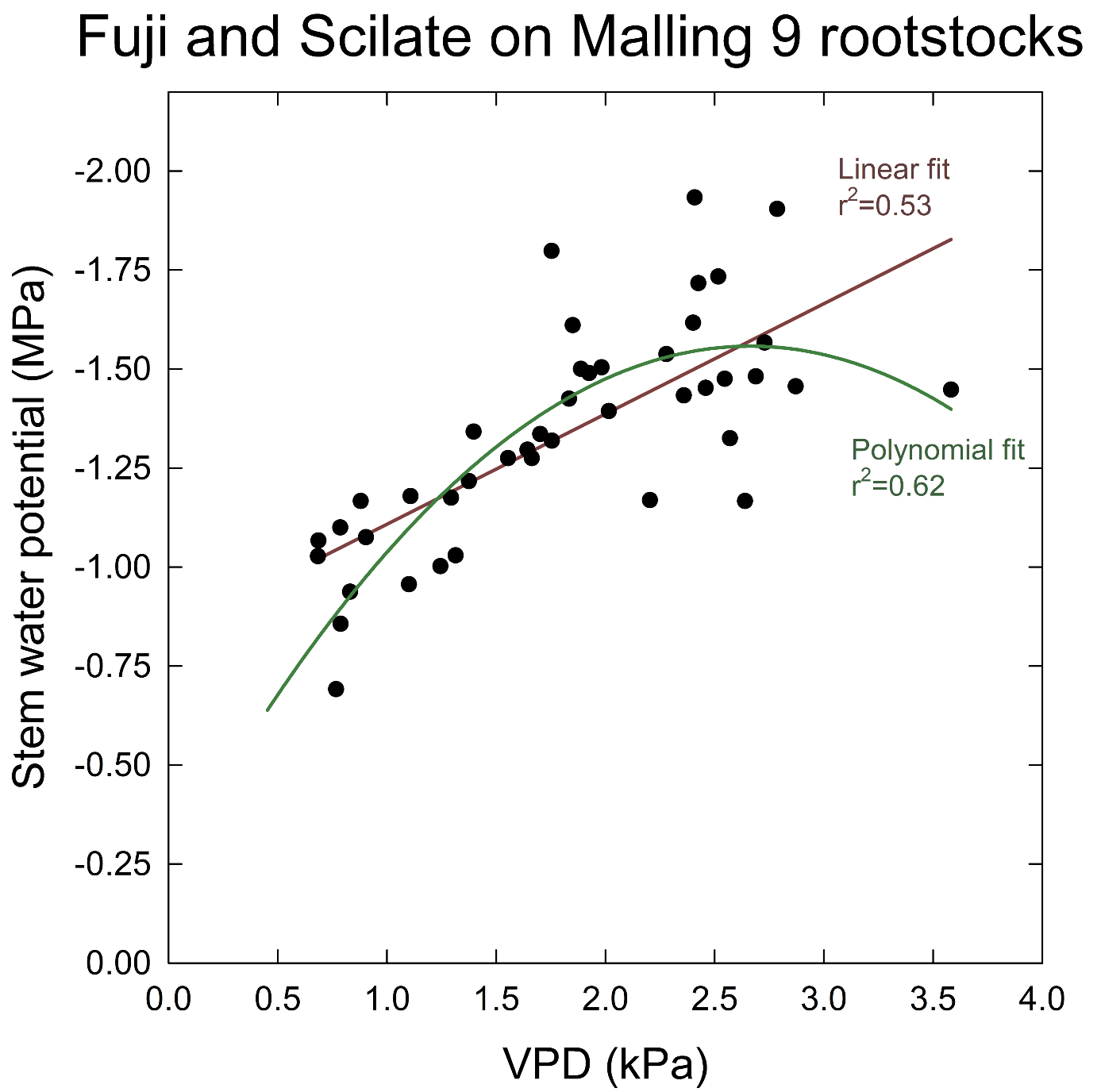


Supplementary Figure 4. Linear and polynomial fit models of vapor pressure deficit (VPD) to stem water potential in two varieties of apple on dwarfing rootstocks. Average daily values of stem water potential from ‘Aztec Fuji’ and ‘Scilate’ (Envy™) on Malling 9 rootstocks in relation to VPD. Saturating responses of season long stem water potential to VPD may be better modeled using a polynomial function. Error bars omitted for clarity, n=8.
